# Increased cellular protein modification by methylglyoxal activates endoplasmic reticulum-based sensors of the unfolded protein response

**DOI:** 10.1016/j.redox.2024.103025

**Published:** 2024-01-05

**Authors:** Mingzhan Xue, Zehra Irshad, Naila Rabbani, Paul J. Thornalley

**Affiliations:** a Diabetes Research Center, Qatar Biomedical Research Institute, Hamad Bin Khalifa University (HBKU), Qatar Foundation, P.O. Box 34110, Doha, Qatar; b Clinical Sciences Research Laboratories, Warwick Medical School, University of Warwick, University Hospital, Coventry, CV2 2DX, UK; c Department of Basic Medical Science, College of Medicine, QU Health, Qatar University, P.O. Box 2713, Doha, Qatar; d College of Health and Life Sciences, Hamad Bin Khalifa University, Qatar Foundation, P.O. Box 34110, Doha, Qatar

**Keywords:** Unfolded protein response, ER stress, Hyperglycemia, Methylglyoxal, Glycation, Glycemic disease

## Abstract

The unfolded protein response (UPR) detects increased misfolded proteins and activates protein refolding, protein degradation and inflammatory responses. UPR sensors in the endoplasmic reticulum, IRE1α and PERK, bind and are activated by proteins with unexpected surface hydrophobicity, whereas sensor ATF6 is activated by proteolytic cleavage when released from complexation with protein disulfide isomerases (PDIs). Metabolic dysfunction leading to the formation of misfolded proteins with surface hydrophobicity and disruption of ATF6-PDI complexes leading to activation of UPR sensors remains unclear. The cellular concentration of reactive dicarbonyl metabolite, methylglyoxal (MG), is increased in impaired metabolic health, producing increased MG-modified cellular proteins. Herein we assessed the effect of high glucose concentration and related increased cellular MG on activation status of IRE1α, PERK and ATF6. Human aortal endothelial cells and HMEC-1 microvascular endothelial cells were incubated in low and high glucose concentration to model blood glucose control, with increase or decrease of MG by silencing or increasing expression of glyoxalase 1 (Glo1), which metabolizes MG. Increased MG induced by high glucose concentration activated IRE1α, PERK and ATF6 and related downstream signalling leading to increased chaperone, apoptotic and inflammatory gene expression. Correction of increased MG by increasing Glo1 expression prevented UPR activation. MG modification of proteins produces surface hydrophobicity through arginine-derived hydroimidazolone MG-H1 formation, with related protein unfolding and preferentially targets PDIs and chaperone pathways for modification. It thereby poses a major challenge to proteostasis and activates UPR sensors. Pharmacological decrease of MG with Glo1 inducer, *trans*-resveratrol and hesperetin in combination, offers a novel treatment strategy to counter UPR-related cell dysfunction, particularly in hyperglycemia associated with diabetes.

## 1 Introduction

The unfolded protein response (UPR) is a transcriptional and translational signalling network providing surveillance of protein quality and folding. It has three endoplasmic reticulum (ER)-based protein sensors which control different aspects of UPR coordinated proteostasis: inositol requiring enzyme-1α (IRE1α), double-stranded RNA-dependent kinase-like ER kinase (PERK) and activating transcription factor-6 (ATF6). Activation of these sensors is called ER stress [1]. ER stress is a feature of impaired metabolic health – increased fasting and postprandial glucose, and diabetes [2]. It is involved in hyperglycemia-induced dysfunction of endothelial cells (ECs) and development of diabetic vascular disease [3]. Increased plasma glucose concentration induces EC dysfunction characterized by increased inflammatory cytokine secretion, expression of adhesion molecules and apoptosis linked to the chronic development of vascular disease [[4], [5], [6], [7]].

The major types of misfolded proteins and chemical biology of their formation that activate UPR sensors in the physiological setting remains unclear. Experimental studies of ER stress have often used pharmacological UPR activators such as tunicamycin, an inhibitor of enzymatic protein glycosylation [8], although depleted enzymatic protein glycosylation is not required to activate the UPR physiologically.

IRE1α is an auto-phosphorylating kinase which, on activation, cleaves X-box-binding protein 1 (XBP1) mRNA to form splice variant transcription factor, XBP1s. Expression of the unspliced variant XBP1u occurs concurrently and is a negative regulator of XBP1s [9]. XBP1s increases expression of ER chaperones, genes of ER-associated protein degradation (ERAD), lipid synthesis and protein disulfide isomerases (PDIs) [[10], [11], [12]]. PERK is also an auto-phosphorylating kinase which phosphorylates eukaryotic translation initiation factor-2α (eIF2α) leading to temporary inhibition of protein translation [13] with a subset of mRNAs translationally induced, including ATF4, controlling expression of genes of amino acid metabolism, redox homeostasis and C/EBP homologous protein (CHOP)-regulated apoptotic signalling [14]. ATF6 is a transcription factor regulating expression of ER chaperones, ERAD, PDIs and other ER proteins. When the UPR sensors detect an abnormally high level of misfolded proteins, there is increased expression of chaperones and ubiquitin ligases to refold or degrade misfolded proteins, a temporary stall of translation to inhibit protein synthesis whilst protein refolding is improved, activation of apoptosis where normal proteostasis cannot be regained, and stimulation of low grade inflammation to increase host immunity [1].

IRE1α is activated by misfolded proteins binding to a hydrophobic groove in the luminal domains [15]. Misfolded protein bound IRE1α dimers associate into larger oligomers, enabling autophosphorylation and endoribonuclease activity [16]. The 78-kDa glucose-regulated protein (GRP78) binds close to the transmembrane domain, adjusting sensitivity to misfolded proteins [17,18]. PERK is activated by autophosphorylation and oligomerization which is stimulated on dissociation from ER chaperones GRP78 and 94-kDa glucose-regulated protein (GRP94) – the latter is induced by accumulating misfolded proteins [19]. Misfolded proteins bind a conserved hydrophobic groove of the luminal domain of PERK through interaction with a surface hydrophobic site exposed or characteristic of the misfolded form [20]. ATF6 is activated by translocation to the Golgi and therein undergoes successive proteolytic cleavage site-1 and site-2 proteases (S1P and S2P, respectively), releasing the active 50 kDa amino-terminal cytoplasmic fragment (ATF6-N) [21].

**Fig. 1:**
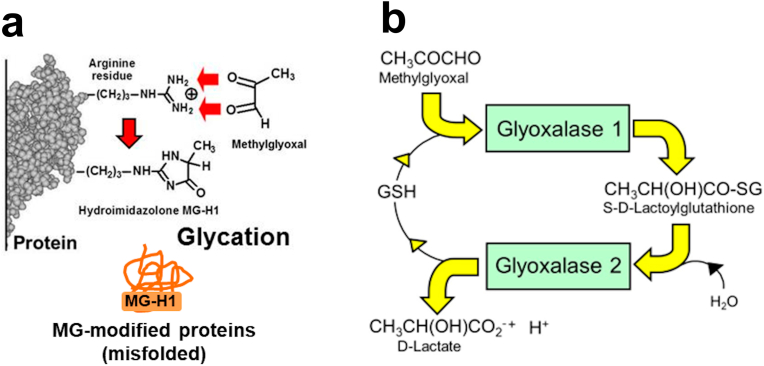
Protein glycation by methylglyoxal and metabolism of methylglyoxal by the glyoxalase pathway. a Protein glycation by methylglyoxal (MG) – formation of hydroimidazolone, N_δ_-(5-hydro-5-methyl-4-imidazolon-2-yl)-ornithine (MG-H1). **b** Metabolism of methylglyoxal by the glyoxalase system.

In high glucose concentration, the cellular concentration of reactive dicarbonyl metabolite, methylglyoxal (MG), was increased in ECs [22,23] – an abnormal metabolic state called dicarbonyl stress [24]. MG modifies proteins to form mainly hydroimidazolone MG-H1 – a hydrophobic moiety formed often on the surface of proteins from hydrophilic arginine residues precursors and induces protein misfolding (Fig. 1a) [25]. MG also preferentially modifies PDIs, particularly PDI-A1, -A3, -A4 and -A6 [22,26]. Increased MG also induced upregulation of heat shock proteins, HSP70, GRP78 and others [22]. MG is mainly metabolized by glyoxalase 1 (Glo1) of the glyoxalase pathway (Fig. 1b).

In this study, we assessed the involvement of dicarbonyl stress in the physiological activation of the UPR – exploring if UPR sensor pathways are activated by high glucose concentration in ECs *in vitro* and if this is mediated by increasing cellular MG concentration. We increase dicarbonyl stress in ECs by siRNA silencing of Glo1 and alleviate it by increasing Glo1 expression through vector expression and optimised small molecule Glo1 expression inducer, *trans*-resveratrol and hesperetin in combination (tRES + HESP) [27]. We also assessed how pharmacological activation of the UPR by tunicamycin in ECs compares to the response to high glucose concentration.

## 2 Materials and methods

### 2.1 Reagents

Human aortal endothelial cells (HAECs), certified mycoplasma-free, and culture reagents, Endothelial Cell Growth Medium-2 (EGM™-2) Bullet Kit™ Culture system containing EBM™-2 Endothelial Basal Medium (CC-3156), and EGM™-2 Single Quots™ supplements, were purchased from Lonza, Basel, Switzerland. The HMEC-1 microvascular endothelial cell line, certified mycoplasma-free, was purchased from ScienCell, Carlsbad, USA.

Antibodies used in Western blotting were: rabbit polyclonal anti-human Glo1 (prepared in-house [28]; rabbit polyclonal anti-human EIF2α (Cat no 9722), anti-human phospho-EIF2α [Ser51] (Cat no 9721) and anti-human beta-actin (Cat no 4967), rabbit monoclonal anti-human IRE1α, clone 14C10 (Cat no 3294), anti-human PERK, clone C33E10 (Cat no 3292), anti-human phospho-PERK [Thr980] (Cat no 3179) and anti-human TXNIP, clone D5F3E (Cat no 14715), and mouse monoclonal anti-human CHOP (Cat no 2895) purchased from Cell Signalling Technology (Danvers, MA, USA); rabbit monoclonal anti-human phospho-IRE1α [S724] (Cat no ab48187) and anti-human ATF6 (Cat no ab227830) purchased from Abcam (Cambridge, UK); rabbit polyclonal anti-human XBP1 (Cat no PA5-95260) purchased from ThermoFisher (Waltham, MA, USA); and goat polyclonal anti-rabbit IgG, HRP conjugate (Cat no 32260) and anti-mouse IgG, HRP conjugate (Cat no 31430) purchased from Invitrogen (Waltham, MA, USA).

Oligonucleotides used this study were: ON-TARGETplus SMART pool human GLO1 siRNA (Cat no L-012277-00-0010), ON-TARGETplus SMART pool human XBP1 siRNA (Cat no L-009552-00-0010), miRIDIAN microRNA Human hsa-miR-17-5p - Mimic (Cat no C-300485-05-0010), and miRIDIAN microRNA human hsa-miR-17-5p - hairpin inhibitor (Cat no IH-300485-06-0010), purchased from Dharmacon (Lafayette, CO, USA).

Expression vectors used for Glo1 overexpression and empty vector control were pIRES2-GLO1-EGFP and pIRES-EGFP – prepared in-house as described [29].

Chemicals used were: IRE1α inhibitor, 7-hydroxy-4-methyl-2-oxo-2H-1-benzopyran-8-carboxaldehyde 4μ8C and hesperetin, purchased from Merck, Darmstadt, Germany (Cat no SML-0949 and W431300, respectively); tunicamycin, purchased from Cell Signalling Biotechnology (Cat no 12819S); Lipofectamine™ RNAiMAX Transfection Reagent, purchased from ThermoFisher (cat no 13778500); and *tran*s-resveratrol, purchased from Cayman Chemical Company, Ann Arbor, MI, USA (Cat no 70675).

### 2.2 Cell culture

HAECs were cultured in Endothelial Cell Growth Medium-2 (EGM™-2) Bullet Kit™ Culture system. They were studied during passages 4–6 during which the primary aortal endothelial phenotype is maintained. HMEC-1 microvascular endothelial cell line was cultured in MCDB-131 medium supplemented with 10 % fetal bovine serum, 10 mM L-glutamine, 10 ng/mL epidermal growth factor, 1 μg/mL hydrocortisone, 100 units/ml penicillin and 100 μg/mL streptomycin [5]. Both HAEC and HMEC-1 cultures were maintained in 100 % humidified environment at 37 °C with 5 % CO_2_. Other culture conditions were: low glucose (4.1 mM d-glucose, LG) and high glucose (20 mM d-glucose, HG). Cell viability was assessed by the Trypan blue exclusion method.

For Glo1 and XBP1 knockdown studies, 2–8 × 10^5^ of HAECs were transfected with 25 nM target Accell Human SMART siRNA pool or an Accell non-targeting Control siRNA pool with Lipofectamine® *RNAiMAX* Transfection Reagent; after 24 h, the cells were treated with LG and HG with or without other additions for 72 h, unless otherwise stated. RNA and protein were then extracted and stored at −80 °C for further analysis. Other additions were: 1 μM 4μ8C and 5 μM tRES + HESP. The study was approved by HBKU Institutional Biosafety Committee (Project no. 2019/022).

### 2.3 Western blotting

Cells were collected by trypsinization and centrifugation, cell pellets lysed, and protein extracted with RIPA Lysis and Extraction buffer (ThermoScientific, 25 mM Tris/HCl pH 7.6, 150 mM NaCl, 1 % NP-40, 1 % sodium deoxycholate, 0.1 % SDS) and supplemented with protease and phosphatase inhibitors (Thermo Fisher Scientific). The protein concentration was determined by the detergent compatible (DC) method. Aliquots of protein (20 μg) were separated by SDS-polyacrylamide gel electrophoresis and then transferred to PVDF membranes. Membranes were then blocked with 3 % bovine serum albumin (BSA) in TBST (Tris-buffered saline–Tween buffer,10 mmol/l Tris-HCl, pH 7.5; 150 mmol/l NaCl; and 0.05 % Tween-20) and incubated with indicated primary antibodies overnight at 4 °C with shaking. After washing with TBST, the membrane was incubated with corresponding horseradish peroxidase-linked secondary antibodies at room temperature for 1 h. Both primary and secondary antibodies were diluted with 1 % BSA in TBST. The immunoblotting signal was detected using a chemiluminescence detection kit and band intensities were quantified using ImageQuant TL software (GE Healthcare).

### 2.4 RNA interference and miR-17 agonism and inhibition

siRNAs for targeting human GLO1 and XBP1, ON-TARGETplus human siRNA SMART pool were used to knockdown target genes in HAEC cells. The siRNA sequences are listed in Table S1 siRNA (25 nM) was used to transfect cells with Lipofectamine RNAiMAX following the manufacturer's instruction. The level of mRNA and protein was assessed at 72 h. Human hsa-miR-17-5p mimic and hairpin inhibitor were used following the manufacturer's transfection protocol.

### 2.5 Vector transfection

Plasmid transfection studies for overexpression of Glo1 were performed with HMEC-1 cells. Transfection of plasmid pIRES2-GLO1-EGFP for Glo1 overexpression used FuGENE HD transfection reagent (Promega, Madison, WI, USA; Cat no E2311) following the manufacturer's transfection protocol. For 100 mm dishes, Glo1 overexpression plasmid (8 μg) and 24 μl FuGENE transfection reagent were diluted separately in 500 μl Opti-MEM Reduced Serum Media. The solutions were kept at room temperature for 5 min. Plasmid and FuGENE solutions were then combined, mixed well and kept at room temperature for 15 min. An aliquot of mix FuGENE/plasmid (1 ml) was added to HMEC-1 cells and cultures continued with LG and HG for 72 h. For empty vector control, cells were transfected with empty plasmid pIRES2-EGFP by the same protocol. Plasmids were produced in-house, as described [29].

### 2.6 Relative quantification of mRNA

mRNA of target genes was detected with SYBR green RT-PCR. Total RNA was extracted from cells with the PureLink RNA Mini Kit (Invitrogen™). The quality and quantity of RNA were determined with Nanodrop 2000 spectrophotometer. Aliquots of RNA (500 ng) were used to generate cDNA using the High-Capacity cDNA Reverse Transcription Kit (Applied Biosystems™, 4,368,814). PowerUp™ SYBR™ Green Master Mix SYBR Green (Applied Biosystems™ A25741) was used for qPCR reactions, and the expression of target genes were normalized to housekeeping gene, ribosomal protein lateral stalk subunit P0 (RPLP0). RT-PCR were performed on a QuantStudio™ 7 Flex Real-Time PCR System (Applied Biosystems, Waltham, MA, USA). Relative mRNA expression level of target genes was determined using delta-delta CT (*ddCt*) and presented as fold-change relative to the control. The primers used for RT-PCR are listed in Table S2.

### 2.7 Assay of methylglyoxal and protein glycation and oxidation adducts

The MG content of HMEC-1 cells was determined by stable isotopic dilution analysis liquid chromatography-tandem mass spectrometry (LC-MS/MS) [30]. The glycation and oxidation adduct residues of cytosolic protein extracts and related free adducts in culture medium were determined by stable isotopic dilution analysis liquid chromatography-tandem mass spectrometry [31]. The content of glycation and oxidation adducts in extracts of cellular protein are given as mmol/mol amino acid modified (arg, lys, tyr and trp). The flux of protein glycation and oxidation free adduct formation was deduced by analysis of culture media samples at baseline and end of the culture and deducing the increase in amount of protein glycation and oxidation free adducts in nmol/10^6^ cells/day [32].

### 2.8 Statistical analyses and reproducibility

Data are mean ± SD of a minimum of 3 independent biological replicates and responses were validated for HAECs in cells from at least 3 different donors and HMEC-1 cells from at least 3 difference batches. Significance of difference was assessed by Student's *t-test* (2 groups) and ANOVA (4 groups).

## 3 Results

### 3.1 High glucose concentration activates multiple pathways of the endothelial cell UPR *in vitro* through involvement of dicarbonyl stress

**Fig. 2:**
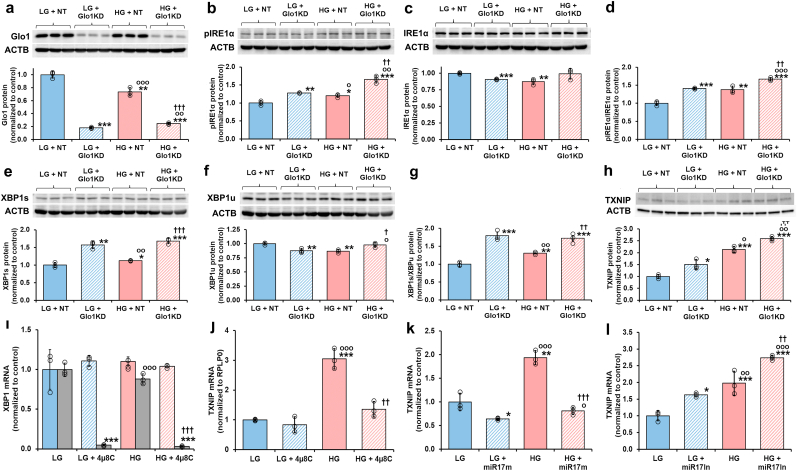
Effect of dicarbonyl stress and high glucose concentration on activation of the IRE1α sensor pathway of the UPR in human aortal endothelial cells *in vitro*. Effect of Glo1 silencing – Western blotting: **a** Glo1, **b** pIRE1α, **c** total IRE1α, **d** pIRE1α/total IRE1α ratio (ratio of bands in **b** and **c**), **e** XBP1s, **f** XBP1u, **g** XBP1s/XBP1u ratio (ratio of bands in **e** and **f**), and **h** TXNIP. Key: LG + NT, low glucose concentration (4.1 mM) + non-target siRNA; LG + Glo1KD, low glucose concentration + Glo1 siRNA (knockdown); HG + NT, high glucose concentration (20 mM) + non-target siRNA; and HG + Glo1KD, high glucose concentration + Glo1 siRNA. **i** and **j** Effect of IRE1α inhibitor, 4μ8C, on XBP1 mRNA splicing and expression of TXNIP, respectively. Effect of miR-17 agonism and inhibition on expression of TXNIP: **k** TXNIP mRNA with and without miR-17 mimic (miR17m); and **l**, TXNIP mRNA with and without miR-17 inhibitor (miR17In). Key: LG, low glucose concentration (4.1 mM); LG+4μ8C/miR17m/miR17In, low glucose concentration + 4μ8C, miR-17 mimic or miR-17 inhibitor; HG, high glucose concentration; HG+4μ8C/miR17m/miR17In, high glucose concentration + 4μ8C, miR-17 mimic or miR-17 inhibitor. Data are mean ± SD (n = 3). Significance: *, ** and ***, *p* < 0.05, *p* < 0.01 and *p* < 0.001 with respect to LG or LG + NT control; o, oo and ooo, *p* < 0.05, *p* < 0.01 and *p* < 0.001 with respect to LG + Glo1KD, 4μ8C, miR-17 m or miR-17In; and †, †† and †††, *p* < 0.05, *p* < 0.01 and *p* < 0.001 with respect to HG or HG + NT control (*Student's t-test*). *ANOVA*: *p* < 0.001 except for **c** (P < 0.05) and **f** (P < 0.01). Incubations were for 72 h. Key to bar shading: solid pastel blue and red bars, low and high glucose concentration controls, respectively; pastel blue and red bars hatched bars, low and high glucose concentration with further additions, respectively; and grey bars in **i**, XBP1s mRNA. Abbreviation: ACTB, β-actin housekeeping protein. (For interpretation of the references to colour in this figure legend, the reader is referred to the Web version of this article.)

Dicarbonyl stress is induced by high glucose concentration (HG) in human aortal endothelial cells (HAECs) *in vitro* through increase of MG formation concomitant with hexokinase-2-dependent increased glucose metabolism and modest decrease in Glo1 (due to metabolic dysfunction linked increased proteolysis) [22]. Cellular MG concentration was 2.22 ± 0.56 pmol/10^6^ cells and increased *ca.* 2-fold in HG (Table 1). This increase may recapitulated in low glucose concentration (LG) by Glo1 knockdown and the cellular MG concentration increased further by 3-fold by GLO1 knockdown in HG [6]. Herein, Glo1 protein was decreased 74 % by Glo1 siRNA silencing in LG (Fig. 2a) - as reported previously [6,22]. In HG, Glo1 protein was decreased 17 %. Glo1 was decreased further by 80 % with Glo1 siRNA silencing (Fig. 2a),

**Table 1:** Increased cellular methylglyoxal and protein glycation and oxidation in human aortal endothelial cells in high glucose concentration.

Analyte	Culture conditions	
	LG	HG
**Cellular methylglyoxal**		
MG (pmol/10^6^ cells)	2.22 ± 0.56	4.85 ± 0.56***
**Cellular protein glycation and oxidation adducts**		
FL (mmol/mol lys)	2.92 ± 1.60	5.71 ± 1.05**
CML (mmol/mol lys)	0.200 ± 0.113	0.256 ± 0.150
CEL (mmol/mol lys)	0.032 ± 0.016	0.046 ± 0.024
CMA (mmol/mol arg)	0.013 ± 0.004	0.012 ± 0.003
MG-H1 (mmol/mol arg)	0.417 ± 0.133	0.604 ± 0.095*
DT (mmol/mol tyr)	0.151 ± 0.064	0.146 ± 0.065
NFK (mmol/mol trp)	0.394 ± 0.264	0.251 ± 0.085
**Flux of formation of protein glycation and oxidation adduct**s **(pmol/10**^**6**^ **cells/day)**		
FL	164 ± 42	342 ± 74 ***
CML	8.11 ± 3.40	22.85 ± 6.50***
CEL	10.0 ± 1.8	18.1 ± 6.5***
CMA	49.5 ± 10.3	51.1 ± 3.2
MG-H1	14.1 ± 3.7	24.0 ± 3.2***
DT	13.0 ± 3.0	17.3 ± 5.0
NFK	14.3 ± 3.6	13.5 ± 3.7

Data are mean ± SD (n = 6). Significance: *, ** and ***, *p* < 0.05, *p* < 0.01 and *p* < 0.001 with respect to Control (*t*-test). Protein glycation and oxidation adduct data are for HAEC cultures (LG = 5 mM glucose and HG = 20 mM glucose). MG content data are for HMEC-1 endothelial cells (LG = 5 mM glucose and HG = 30 mM glucose). Cultures were for 6 days.

UPR sensor IRE1α pathway activation status was assessed by autophosphorylation of IRE1α on S724 (pIRE1α). pIRE1α was increased 20 % in HG, with a similar increase produced by silencing of Glo1 in LG and further increased by 67 % with Glo1 silencing in HG (Fig. 2b). Total IRE1α protein was decreased modestly, 13 %, in HG – again, an effect also produced by silencing of Glo1 in LG (Fig. 2c). We interpret this an auto-regulatory IRE1α switch-off response by downregulation of IRE1α expression. This may be produced by regulated IRE1α-dependent decay (RIDD) activity, cleaving its own mRNA [33], and by effects of increased XBP1s and ATF6 (see below) which also downregulate expression of IRE1α [34,35]. PDI-A6 also limits the duration of IRE1α activation by direct binding to cysteine 148 in the luminal domain of the sensor, which is oxidized when IRE1 is activated [36] and decreasing RIDD activity [37]. The pIRE1α/total IRE1α protein ratio, an indication of the proportion of IRE1α sensors activated, was increased 38 % in HG, recapitulated by Glo1 silencing in LG and increased further by Glo1 silencing in HG (Fig. 2d).

Immediate downstream effects of IRE1α activation is increased expression of XBP1s [38]. XBP1s protein was increased 13 % by HG and increased 57 % and 68 % with Glo1 silencing in LG and HG, respectively (Fig. 2e). XBP1u protein was decreased 13 % in HG, with a similar effect produced by Glo1 silencing in LG (Fig. 2f). XBP1s/XBP1u protein ratio was increased 30 % in HG and by 80 % and 72 % with Glo1 silencing in LG and HG respectively (Fig. 2g).

Also immediately downstream of IRE1α activation is increased expression of thioredoxin interacting protein (TXNIP) through RIDD of microRNA-17 (miR-17) which otherwise destabilizes TXNIP mRNA [38]. TXNIP links activation of the UPR to the nucleotide-binding domain, leucine-rich–containing family, pyrin domain–containing-3 (NLRP3) inflammasome and production of inflammatory mediators in ECs [39]. TXNIP protein was increased by Glo1 knockdown in LG, and progressively increased further in HG and Glo1 knockdown in HG (Fig. 2h). To confirm the role of activation of IRE1α in increased expression of XBP1s and TXNIP, we studied the effect of IRE1α inhibitor, 4μ8C, on splicing of XBP1 mRNA and increase of TXNIP mRNA [11]. Inhibition of IRE1α with 4μ8C blocked the formation of spliced XBP1 mRNA (XBP1s) in LG and HG cultures (Fig. 2i, grey bars) and blocked the increase of TXNIP mRNA in HG cultures (Fig. 2j). Therefore, the formation of both XBP1s and the increase of TXNIP expression in HG occurs by the endoribonuclease activity of IRE1α.

We sought further evidence of involvement of miR-17 in increased expression of TXNIP by IRE1α activated by HG in ECs. Addition of miR-17 mimic decreased TXNIP mRNA in LG and completely countered the increase of TXNIP mRNA in HG cultures (Fig. 2k). In contrast, addition of miR-17 inhibitor increased TXNIP mRNA in LG and further increased TXNIP mRNA in HG cultures (Fig. 2l). De-stabilization of TXNIP mRNA by miR-17 therefore has a key influence on expression of TXNIP in ECs and countering of this produces the increase of TXNIP in response to activation of IRE1α in HG.

**Fig. 3:**
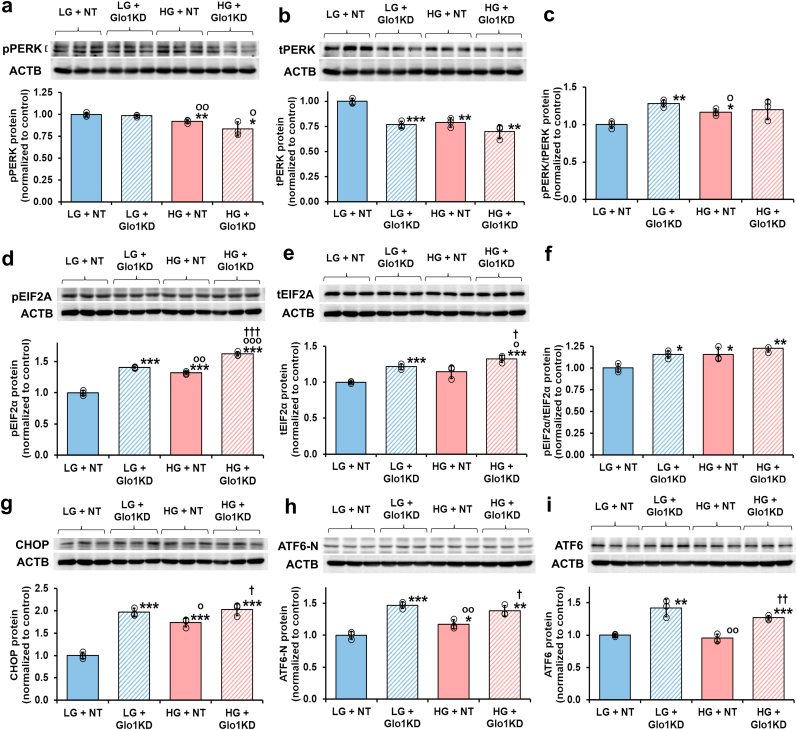
Effect of dicarbonyl stress and high glucose concentration on activation of the PERK and ATF6 sensor pathways of the UPR in human aortal endothelial cells *in vitro*. Effect of Glo1 silencing – Western blotting: **a** pPERK, **b** tPERK, **c** pPERK/tPERK ratio (ratio of bands in **a** and **b**), **d** pEIF2α, **e** tEIF2α, **f** pEIF2α/tEIF2α ratio (ratio of bands in **d** and **e**), **g** CHOP, **h** ATF6-N, and **i** ATF6. Key: LG + NT, low glucose concentration (4.1 mM) + non-target siRNA; LG + Glo1KD, Low glucose concentration + Glo1 siRNA (knockdown); HG + NT, high glucose concentration (20 mm) + non-target siRNA; and HG + Glo1KD, high glucose concentration + Glo1 siRNA. Significance: *, ** and ***, *p* < 0.05, *p* < 0.01 and *p* < 0.001 with respect to LG + NT control; o, oo and ooo, *p* < 0.05, *p* < 0.01 and *p* < 0.001 with respect to LG + siRNA; and †, †† and †††, *p* < 0.05, *p* < 0.01 and *p* < 0.001 with respect to HG + NT control (*Student's t-test*). *ANOVA*: *p* < 0.001 except for **a** and **f** (*p* < 0.01) and **c** (p < 0.05). Key to bar shading: solid pastel blue and red bars, low and high glucose concentration controls, respectively; and pastel blue and red bars hatched bars, low and high glucose concentration with further additions, respectively. Abbreviation: ACTB, β-actin housekeeping protein. (For interpretation of the references to colour in this figure legend, the reader is referred to the Web version of this article.)

We assessed the activation status of the PERK pathway in HAECs by HG. PERK activation was judged by the ratio of PERK protein phosphorylated in the activation loop on thr-980 (pPERK) to total PERK protein (pPERK/tPERK) [19]. pPERK was modestly decreased 8 % in HG and 17 % in HG with Glo1 silencing (Fig. 3a) whereas total PERK protein was decreased 23–30 % in HG and in LG and HG with Glo1 silencing (Fig. 3b), with the related pPERK/tPERK ratio increased 20–28 % (Fig. 3c). Therefore, an increased proportion of PERK sensor protein is activated in HG and this activation is recapitulated by Glo1 silencing in LG and not increased further by Glo1 silencing in HG.

Immediately downstream in the PERK pathway is phosphorylation of EIF2α on Ser-51 (pEIF2α) which inhibits eIF2β-dependent exchange of GDP for GTP, thereby preventing protein translation [40]. The EIF2α activation status depends on the ratio of pEIF2α to total EIF2α, pEIF2α/tEIF2α. pEIF2α, tEIF2α and pEIF2α/tEIF2α ratio were increased in HG. They were increased similarly by Glo1 silencing in LG and HG (Fig. 3d–f). EIF2α signalling is therefore activated in HG and this activation is recapitulated by Glo1 silencing in LG. Further downstream of EIF2α in the PERK pathway is regulation of expression of CHOP. CHOP protein was increased 74 % in HG and increased similarly by Glo1 silencing in LG and HG (Fig. 3g).

Activation of ATF6 is judged by increased levels of ATF6-N terminal fragment, ATF6-N protein. In LG with Glo1 silencing, ATF6-N protein was increased 47 %. ATF6-N protein was increased 17 % in HG without Glo1 silencing and increased 38 % in HG with Glo1 silencing (Fig. 3h). Interestingly, full length ATF6 protein was increased in LG and HG with Glo1 silencing but not in HG with non-target siRNA (Fig. 3i). The activated PERK pathway increases the synthesis of ATF6 through ATF4-regulated expression and trafficking of ATF6 from the ER to the Golgi for intramembrane proteolysis and activation of ATF6 [41]. Downstream of ATF6 activation, is increased expression of GRP78 [42] which we previously reported was increased in HAECs in HG, with the increase recapitulated by Glo1 silencing in LG and further increased by Glo1 silencing in HG [22].

### 3.2 Overexpression and induction of glyoxalase 1 expression prevents activation of the UPR

We next assessed the importance of dicarbonyl stress in the activation of the UPR in ECs by HG. In our cell culture models, we used two interventions to counter dicarbonyl stress: overexpression of Glo1 by vector transfection or treatment with tRES + HESP [6,27,29].

**Fig. 4:**
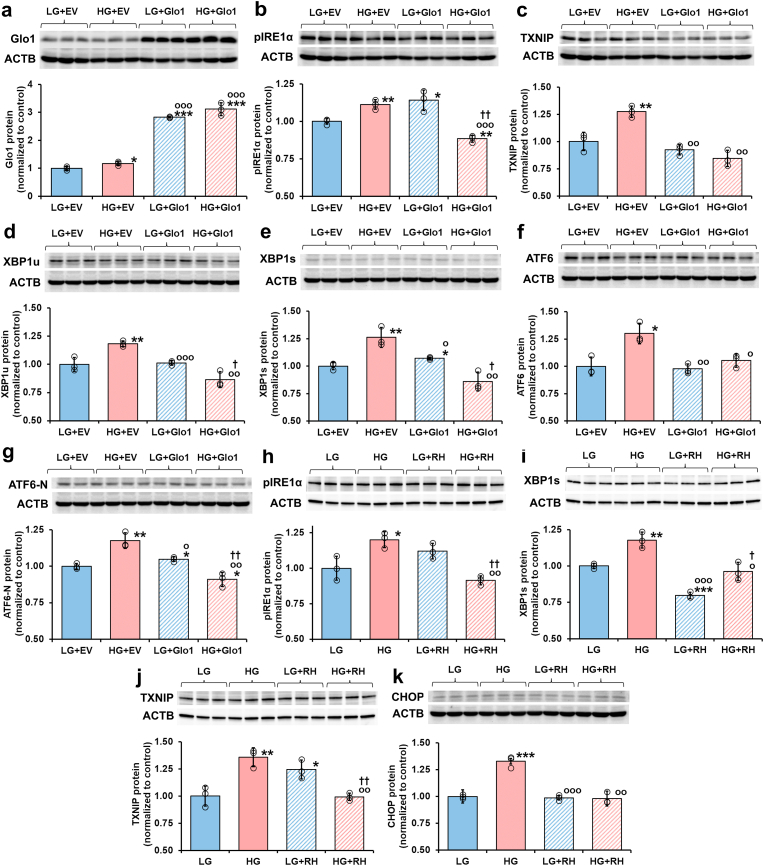
Effect of glyoxalase 1 overexpression and induction by Glo1 inducer, *trans*-resveratrol and hesperetin, on activation of IRE1α and signalling in human endothelial cells *in vitro*. **a - g** Effect of overexpression of Glo1 on activation of the UPR. Western blotting: **a** Glo1, **b** pIRE1α, **c** TXNIP, **d** XBP1u, **e** XBP1s, **f** ATF6 and **g** ATF6-N. **h** – **k** Effect of Glo1 inducer, tRES + HESP, on activation of the UPR. Western blotting: **h** pIRE1α, **i** XBP1s, **j** TXNIP, and **k** CHOP protein. Key: LG, low glucose concentration; LG + EV, low glucose concentration + empty vector; LG + Glo1, low glucose concentration + Glo1 overexpression vector; LG + RH, low glucose concentration +5 μM tRES + HESP; HG, high glucose concentration; HG + EV, high glucose concentration + empty vector; HG + Glo1, high glucose concentration + Glo1 overexpression vector; and HG + RH, high glucose concentration +5 μM tRES + HESP. Data are mean ± SD (n = 3). Significance: *, ** and ***, *p* < 0.05, *p* < 0.01 and *p* < 0.001 with respect to LG + EV or LG control; o, oo and ooo, *p* < 0.05, *p* < 0.01 and *p* < 0.001 with respect to HG + EV or HG control; and †, ††, and †††, *p* < 0.05, *p* < 0.01 and *p* < 0.001 with respect to HG or HG + EV control; and o and oo, *p* < 0.05 and *p* < 0.01 with respect to LG + Glo1 or LG + RH control; (*Student's t-test*). *ANOVA*: P < 0.001 except **h**, *p* < 0.01. Incubations were for 72 h. Key to bar shading: solid pastel blue and red bars, low and high glucose concentration controls, respectively, with or without empty vector transfection; and pastel blue and red bars hatched bars, low and high glucose concentration, respectively, with further additions (Glo1 overexpression vector or 5 μM tRES + HESP), respectively. Abbreviation: ACTB, β-actin housekeeping protein. (For interpretation of the references to colour in this figure legend, the reader is referred to the Web version of this article.)

Overexpression of Glo1 by vector transfection of HMEC-1 cells produced a 3–4 fold increase of Glo1 protein (Fig. 4a). We evaluated the effect of this on UPR sensors pIRE1α and ATF6, and downstream mediators TXNIP, XBP1u, XBP1s, and ATF6-N proteins. All of these proteins were increased in cells incubated in HG with empty vector transfection and all of the increases were corrected to LG empty vector control levels or below by transfection with overexpression of Glo1 (Fig. 4b–g).

Treatment of HAECs with Glo1 inducer, tRES + HESP, increased Glo1 expression and activity of Glo1 by *ca.* 2-fold [22]. We evaluated the effect of this on UPR sensor pIRE1α and downstream mediators XBP1s, TXNIP, and CHOP proteins. All of these proteins were increased in cells incubated in HG and all increases were corrected to levels in LG control cultures by treatment with Glo1 inducer (Fig. 4h–k).

These effects of the two interventions to increase Glo1 expression and suppress dicarbonyl stress suggest this is an effective strategy to correct activation of the UPR in HG. It also indicates that increased cellular MG is a major activator of the UPR in hyperglycemia.

### 3.3 Inflammatory signalling in the endothelial cell UPR stimulated by high glucose concentration – effects of TXNIP and XBP1

**Fig. 5:**
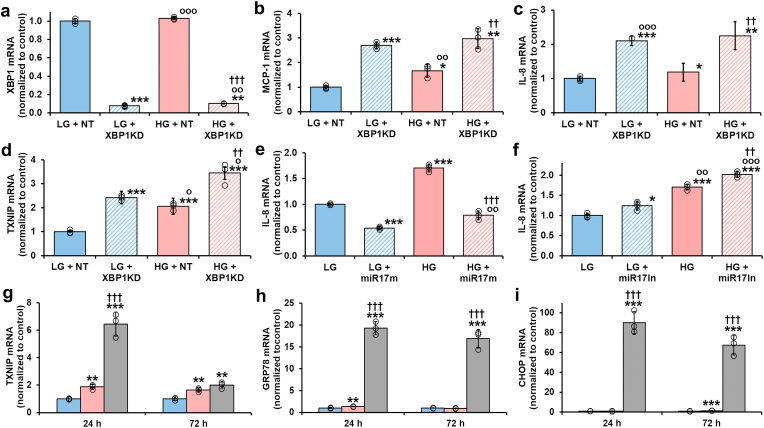
Increased TXNIP and inflammatory signaling in high glucose concentration-induced ER stress in human aortal endothelial cells *in vitro*. Effect of XBP1 knockdown: **a** XBP1 mRNA, **b** MCP-1 mRNA, **c** IL-8 mRNA, and **d** TXNIP mRNA. Key: LG + NT, low glucose concentration + non-target siRNA; LG + XBP1KD, low glucose concentration + XBP1 siRNA; HG + NT, high glucose concentration + non-target siRNA; and HG + XBP1KD, high glucose concentration + XBP1 siRNA. Effect of miR-17 activity on inflammatory signalling: **e** Effect of miR-17 mimic on TXNIP mRNA; and **f** miR-17 inhibitor on TXNIP mRNA. Comparison of UPR-linked gene expression induced by high glucose concentration and treatment with Tunicamycin: **g** TXNIP mRNA, **h** GRP79 mRNA, and **i** CHOP mRNA at 24 h and 72 h, as indicated. Key: LG, low glucose concentration control; LG + NT, low glucose concentration + non-target siRNA control; LG + XBP1KD, low glucose concentration + XBP1 siRNA (knockdown); LG + miR17 m or miR17In, low glucose concentration + miR-17 mimic or inhibitor; HG, high glucose concentration; HG + NT, high glucose concentration + non-target siRNA; HG + XBP1KD, high glucose concentration + XBP1 siRNA (knockdown); and HG + miR17 m or miR17In, high glucose concentration + miR-17 mimic or inhibitor. Data are mean ± SD (n = 3). Significance: *, ** and ***, *p* < 0.05, *p* < 0.01 and *p* < 0.001 with respect to LG or LG + NT control; o, oo and ooo, *p* < 0.05, *p* < 0.01 and *p* < 0.001 with respect to LG + XBP1KD, LG + miR17 m or LG + miR17In; and †† and †††, *p* < 0.01 and *p* < 0.001 with respect to HG or HG + NT control (*Student's t-test*). *ANOVA*: all P < 0.001. Incubations were for 72 h. Key to bar shading: solid pastel blue and red bars, low and high glucose concentration controls; pastel blue and red bars hatched bars, low and high glucose concentration with further additions, respectively; and grey bar, + Tunicamycin. (For interpretation of the references to colour in this figure legend, the reader is referred to the Web version of this article.)

The UPR is an important regulator of inflammatory genes in ECs [43]. IRE1α-dependent RIDD-mediated increased expression of TXNIP increases expression of inflammatory mediators through activation of the NLRP3 inflammasome [44] whereas basal expression of XBP1 in ECs had anti-inflammatory effects [45]. We explored if these responses may be related – particularly pertinent as XBP1 mRNA and miR-17 are competing substrates for endoribonuclease activity of pIRE1α. We decreased expression of XBP1 by siRNA silencing, decreasing XBP1 mRNA by >90 %, and assessed mRNA levels of monocyte chemoattractant protein-1 (MCP-1), interleukin-8 (IL-8) and TXNIP. All these inflammatory mediators were increased in HG. Remarkably, silencing of XBP1 increased the expression of MCP-1, IL-8 and TXNIP in LG cultures and potentiated the increases of these inflammatory mediators in HG cultures (Fig. 5a–d). This indicates that XBP1 has an anti-inflammatory function in HAECs in LG and HG culture conditions.

Pro-inflammatory signaling in HG cultures may therefore occur via TXNIP-NLRP3 inflammasome signalling. To explore this, we studied the effect of miR-17 mimic and inhibition on effect of the expression of IL-8 – an inflammatory marker increased in HG cultures of HAECs and corrected by treatment with tRES + HESP [22]. miR-17 mimic decreased IL-8 mRNA levels of HAECs in LG and corrected the increase of IL-8 mRNA levels of HAECs in HG (Fig. 5e). Conversely, miR-17 inhibitor had the counter effect: increasing levels of IL-8 mRNA of HAECs in LG and further increasing the elevated levels of IL-8 mRNA of HAECs in HG (Fig. 5f). This indicates that in HAECs in high glucose concentration, TXNIP has a dominant pro-inflammatory role – likely through providing linkage of the UPR to the NLRP3 inflammasome [39,44].

### 3.4 Comparison of activation of the UPR in endothelial cells by tunicamycin with the response to high glucose concentration

Tunicamycin (0.5–10 μg/mL) has been widely used as an activator of the UPR in cell studies [46]. Herein we used a relatively low concentration of tunicamycin, 0.75 μg/mL, and assessed mRNA levels in HAECs at 24 h and 72 h of GRP78, CHOP and TXNIP - markers of chaperone, apoptosis and inflammatory signalling, respectively. Whilst tunicamycin induced an increase of TXNIP mRNA like that found in HG, increased 2-fold at 72 h (Fig. 5g), it induced markedly higher expression of GRP78 and CHOP than HG; *cf*. GRP78 mRNA at 24 h, 34 % increase in HG and 19-fold increase with tunicamycin, and CHOP mRNA at 72 h, 43 % increase in HG and 90-fold increase with tunicamycin (Fig. 5h and i). This indicates that tunicamycin induces a similar pro-inflammatory response, but profoundly increased ER chaperone and apoptotic signalling response compared to high glucose concentration.

### 3.5 Protein glycation and oxidation adducts of human aortal endothelial cells and effect of high glucose concentration

We quantified the content of early-stage glucose-derived glycation adduct, N_ε_-fructosyl-lysine (FL), and major advanced glycation endproducts (AGEs), hydroimidazolone MG-H1 and N_ε_(1-carboxyethyl)lysine (CEL) - formed from MG, N_ε_-carboxymethyl-lysine (CML) – formed by the degradation of FL and protein glycation by glyoxal, N_ω_-carboxymethyl-arginine (CMA) – formed from protein glycation by glyoxal only, and oxidation adducts, dityrosine (DT) and N′-formyl-kynurenine (NFK) [47,48]. We measured both the content of adduct residues of cell protein and flux of formation of free adducts formed by cellular proteolysis and released into culture medium (Table 1). Several glycation adducts show increased fluxes of formation in HG cultures – FL, CML, MG-H1 and CEL, whereas other glycation and oxidation adducts did not. The lack of increase in flux of formation of DT and NFK suggests the antioxidant reserve of HAECs is likely not overwhelmed in HG. For the glycation adducts with increased flux of formation in HG, only FL and MG-H1 residue content of cellular protein was increased – consistent with increased proteolysis of CML and CEL-modified proteins preventing their accumulation in HG.

## 4 Discussion

Herein we found that dicarbonyl stress induced in ECs by model hyperglycemia activated three sensor pathways of the UPR, with the response recapitulated by Glo1 knockdown in normal glucose concentration and prevented in model hyperglycemia by overexpression of Glo1. Activation of UPR sensors and related effector responses was prevented by vector overexpression of Glo1 and Glo1 inducer, tRES + HESP. This indicates that UPR sensors IRE1α, PERK and ATF6 are activated by increased MG or dicarbonyl stress.

Dicarbonyl stress is a potent activator of the UPR because: (i) MG modifies proteins to form misfolded proteins with the MG-H1 moiety producing abnormal surface hydrophobicity – a key characteristic for binding to UPR sensors, IRE1α and PERK [15,18,20,49]; and (ii) MG preferentially targets PDI-A1, -A3, -A4 and -A6 for modification, likely activating ATF6 and slowing the return to quiescence of activated IRE1α and PERK [36,50]. Additionally, MG targets chaperones for modification [22], impairing chaperone-mediated protein refolding and increasing the abundance of misfolded proteins. Consistent with this is previous findings of upregulation of the heat shock response by increased cellular MG and Golgi to ER protein trafficking supporting ERAD of misfolded proteins and inflammatory signalling linked to dicarbonyl stress in ECs induced by high glucose concentration [6,22,26].

**Fig. 6:**
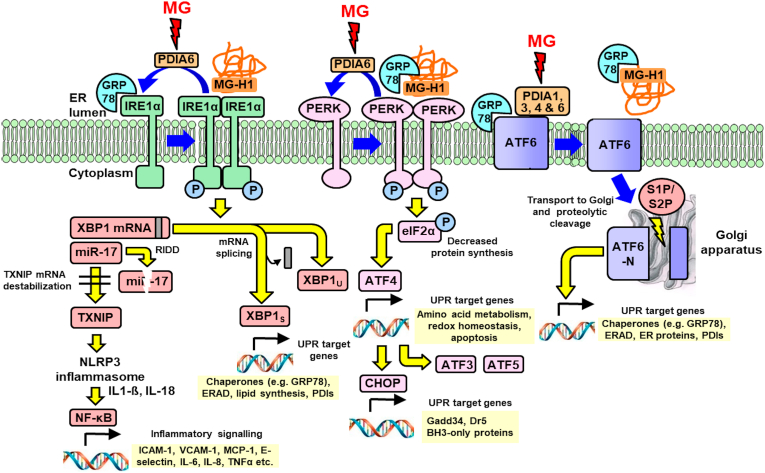
Pathways of the UPR – indicating interactions with methylglyoxal and methylglyoxal-modified proteins. Blue arrows are processes of UPR sensor activation and deactivation; yellow arrows are UPR signalling; and red arrows are PDI modification by MG. Abbreviations: ATF3, ATF4, ATF5 and ATF6, activating transcription factor-3, -4, -5 and -6; BH3, proteins with 3 domains homologous to BCL-2; CHOP, C/EBP homologous protein; Dr5, death receptor-5; eIF2α, eukaryotic translation initiation factor-2α; ER, endoplasmic reticulum; ERAD, endoplasmic reticulum-associated protein degradation; Gadd34, growth arrest and DNA damage-inducible protein; GRP78, 78 kDa glucose-regulated protein; ICAM-1, intercellular adhesion molecule-1; IL-1β, −6, −8 and −18; interleukin-1β, −6, −8 and −18; IRE1α, inositol requiring enzyme-1α; MCP-1, monocyte chemoattractant-1; MG, methylglyoxal; MG-H1, methylglyoxal-derived hydroimidazolone; miR-17, microRNA-17; NLRP3, nucleotide-binding domain, leucine-rich–containing family, pyrin domain–containing-3; P, protein phosphorylation; PDI, protein disulfide isomerase; PERK, double-stranded RNA-dependent kinase-like ER kinase; RIDD, regulated IRE1α-dependent decay; S1P/S2P, site-1 protease/site-2 protease; TNFα, tumor necrosis factor-α; TXNIP, thioredoxin interacting protein; VCAM-1, vascular cell adhesion molecule-1; XBP1, X-box binding protein 1 (subscripts u and s indicate unprocessed mRNA and spliced mRNA expression products, respectively). (For interpretation of the references to colour in this figure legend, the reader is referred to the Web version of this article.)

Activation of IRE1α in HAECs by HG was associated with increased XBP1s/XBP1u ratio, producing increased XBP1 transcriptional activity *in situ* – consistent with the increased abundance of proteins of XBP1s target genes [22]. It was also associated with increased expression of TXNIP, through cleavage of miR-17 by IRE1α stabilizing TXNIP mRNA to degradation, linking UPR activation to the NLRP3 inflammasome. This produces increased expression of IL-8 and other inflammatory mediators [44]. Activation of IRE1α was prevented by overexpression of Glo1 and tRES + HESP induction of Glo1 expression, indicating IRE1α activation is mediated by dicarbonyl stress. Dicarbonyl stress likely also slows the return of activated IRE1α to quiescence, influenced by PDI-A6 [37]. PDI-A6 is a preferential target of MG modification in dicarbonyl stress [26] which may weaken its interaction with IRE1α and support prolonged IRE1α pathway activation in HG (Fig. 6).

The PERK pathway was also activated by dicarbonyl stress in HG, with increased pPERK/tPERK and pEIF2α/tEIF2α ratios and downstream increased expression of CHOP – prevented by Glo1 inducer. MG modification of PDI-A6 may also enhance the activation of PERK [1,36]. Activation of PERK provides a mechanism for regulation of amino acid metabolism, redox homeostasis and apoptotic signalling in ECs in hyperglycemia (Fig. 6).

Proteolytic activation of ATF6 was found in HG where again the effect was replicated by Glo1 silencing in LG, suggesting activation of ATF6 by dicarbonyl stress. ATF6 is stabilized to activatory proteolysis by interaction with PDI-A1, -A3, -A4 and -A6. Chemical modification disrupts this interaction and ATF6 is released for translocation to the Golgi and activation [21]. These PDIs are preferential targets of MG modification [22,26] which may activate ATF6; *cf*. activation of ATF6 by irreversible chemical modification induced by a pharmacological agent [50]. Activated ATF6 produces downstream increased expression of ER chaperones, ERAD, PDIs and other ER proteins (Fig. 6).

Interestingly, both full length ATF6 and ATF6-N were increased markedly with Glo1 silencing in LG and HG, whereas only ATF-N was increased modestly by HG alone (Fig. 3h and i). Increased full-length ATF6 protein likely reflects increased ATF6 expression which is expected from induction through activation of ATF4 in the PERK pathway [41]. High increase of ATF6 expression and ATF6-N signalling response contributes to the switch-off of the activated UPR and return to normal proteostasis [35]. This was a feature of Glo1 silencing in LG and HG, likely reflecting that dicarbonyl stress is a potent activator of the UPR.

As dicarbonyl stress impacts on multiple pathways of the UPR, it may be preferable to target dicarbonyl stress rather than multiple UPR sensor proteins when searching for a druggable target to counter ER stress. Correction of increased MG and prevention of UPR activation offers a new strategy to counter endothelial dysfunction in hyperglycemia associated with diabetes. The health benefits of decreasing dicarbonyl stress and preventing activation of the UPR may extend beyond improved endothelial function and vascular inflammation to countering ER stress in insulin resistance and beta-cell dysfunction in the development of type 2 diabetes [51,52].

An effective strategy to counter activation of the UPR is to induce the expression of Glo1 by tRES + HESP [27]. Treatment of overweight and obese subjects with tRES + HESP for 8 weeks in clinical trial corrected dicarbonyl stress [27]. Therein, decreased expression of TXNIP and inflammatory signalling correlated with improvements in metabolic and vascular health [27,53]. Increased TXNIP contributes to inflammatory signalling in endothelial dysfunction in the development of vascular complications of diabetes [39,54,55], insulin resistance and beta-cell dysfunction [51,52].

It remains uncertain if pharmacological activators of the UPR in experimental studies are a good model of activation of the UPR in hyperglycemia [8]. Tunicamycin produced supra-physiological induction of expression of GRP78 and CHOP [46]. It did not model well the changes in gene expression induced by activation of the UPR in HAECs by HG found herein. Cell responses found in studies with tunicamycin therefore require interpretation with caution when used as a model of effects of hyperglycemia. In future studies, we suggest use of the high glucose concentration model of hyperglycemia and assessing UPR sensor activation status.

From studies of protein glycation and oxidation adducts, the steady-state concentration of both FL and MG-H1 residues were increased in HG. Unlike formation of MG-H1 on arginine residues, FL residue formation on the lysine residue moiety retains the positive charge and gains the hydrophilic 1-deoxyfructosyl moiety. This does not lead to increased surface hydrophobicity in proteins and thereby competency of binding to IRE1α and PERK. Lysine also has lower enrichment in the structured surface functional domains of proteins than arginine (2.1 vs 3.8 fold) [56] which also limits the impact of FL modification on protein structure and folding. Protein modification by MG to form MG-H1 is, therefore, likely an activatory stimulus of UPR sensors because MG-H1 produces unexpected surface hydrophobicity on proteins [25] and MG-H1-modified proteins accumulate in endothelial cells in hyperglycemia.

Glyoxal is also a substrate of Glo1 and precursor of AGEs in ECs and may contribute to UPR activation. The cellular concentration of glyoxal in HAECs is over 10 fold lower than MG [7] and glyoxal is less reactive than MG in protein glycation [57]. The flux of formation of CMA free adduct and cellular protein content of CMA residues were not increased in HAECs in HG (Table 1). This suggests that increase in glyoxal makes a relatively minor contribution to dicarbonyl stress and protein unfolding in endothelial cells in HG.

In summary, UPR sensors, increased MG and MG-modified proteins are major activators of IRE1α, PERK and ATF6 in hyperglycemia. MG modification of proteins produces unexpected surface hydrophobicity and targets PDIs and chaperone pathways – providing a major challenge to proteostasis. Induction of expression of Glo1 offers a new druggable target to counter activation of UPR in diabetes and other dicarbonyl stress-linked disorders [24]. The activation of the UPR by high glucose concentration-induced dicarbonyl stress and health benefits of preventing it likely extends beyond improved endothelial dysfunction and vascular complications of diabetes to countering insulin resistance and beta-cell dysfunction for the prevention of development of type 2 diabetes and related health impairments [53], renal insufficiency and aging where dicarbonyl stress is also common [24]. Indeed, decreased MG-modified protein glycation was recently linked to healthy aging mouse model of ectopic expression of UCP1 in skeletal muscle [58].

## Funding

This research was funded by the 10.13039/100007458Qatar Foundation, grant number QB14 (PJT) and 10.13039/501100004252Qatar University, grant number QU HIG-CMED-2021/22-1 (NR).

## CRediT authorship contribution statement

**Mingzhan Xue:** Investigation, Methodology, Validation, Writing – review & editing. **Zehra Irshad:** Investigation. **Naila Rabbani:** Conceptualization, Funding acquisition, Investigation, Methodology, Supervision, Writing – review & editing. **Paul J. Thornalley:** Conceptualization, Data curation, Formal analysis, Funding acquisition, Investigation, Methodology, Project administration, Resources, Supervision, Validation, Visualization, Writing – original draft.

## Declaration of competing interest

Mingzhan Xue, Naila Rabbani and Paul J Thornalley are co-inventors in the patent of Glo1 inducer, tRES + HESP. Commercial rights to the patent are owned by Glocentrica Ltd (UK). Naila Rabbani and Paul J Thornalley are founding co-directors of Glocentrica Ltd (UK).

## Data Availability

Data will be made available on request.
