# Supplementary material for: Increased cellular protein modification by methylglyoxal activates endoplasmic reticulum-based sensors of the unfolded protein response
Source: Redox Biol. 2024 Jan 5;69:103025. doi: 10.1016/j.redox.2024.103025 (PMC10821617; doi:10.1016/j.redox.2024.103025)
Supplement: Multimedia component 1 [file mmc1.docx]

**Supplementary information -** Sequences of siRNA used for knockdown of target gene expression and primers for PCR.

**Table S1.** Sequences of siRNA used for knockdown of target gene expression.

| Target gene | siRNA sequence |
| --- | --- |
| Human GLO1 | 1. CCUAAGAAGUCACUGGAUU |
|  | 2. GAUGGCUACUGGAUUGAAA |
|  | 3. CUUCUUGGCUUAUGAGGAU |
|  | 4. GAACUGGGAGUCAAAUUUG |
| Human XBP1 | 1. GAACAUCUCCCCAUGGAUU |
|  | 2. ACAGCAAGUGGUAGAUUUA |
|  | 3. CGAAAGAAGGCUCGAAUGA |
|  | 4. GGUAUUGACUCUUCAGAUU |

**Table S2.** Primers for PCR.

| Oligonucleotide target gene (F, forward and R, reverse) | Sequence (5`- 3`) |
| --- | --- |
| XBP1F | CTGCCAGAGATCGAAAGAAGGC |
| XBP1R | CTCCTGGTTCTCAACTACAAGGC |
| CHOPF | TCACCTCCTGGAAATGAAGA |
| CHOPR | TTCTGGCTCCTCCTCAGT |
| HSPA5F (GRP78) | CTGTCCAGGCTGGTGTGCTCT |
| HSPA5R (GRP78) | CTTGGTAGGCACCACTGTGTTC |
| IL8F | GAGAGTGATTGAGAGTGGACCAC |
| IL8R | CACAACCCTCTGCACCCAGTTT |
| GLO1F | ATGAGACCCAGAGTTACCAC |
| GLO1R | CCAGGCCTTTCATTTTACCA |
| CCL2F (MCP-1) | CAGCCAGATGCAATCAATGCC |
| CCL2R (MCP-1) | TGGAATCCTGAACCCACTTCT |
| TXNIPF | CTGGCGTAAGCTTTTCAAGG |
| TXNIPR | AGTGCACAAAGGGGAAACAC |
| RPLP0F | GCAGCATCTACAACCCTGAAG |
| RPLP0R | CACTGGCAACATTGCGGAC |
